# The orphan child: humanities in modern medical education

**DOI:** 10.1186/s13010-018-0067-y

**Published:** 2019-01-04

**Authors:** Mary E. Kollmer Horton

**Affiliations:** Institute for the Liberal Arts, Laney Graduate School, Emory University, Atlanta, Georgia

## Abstract

Use of humanities content in American medical education has been debated for well over 60 years. While many respected scholars and medical educators have purported the value of humanities content in medical training, its inclusion remains unstandardized, and the undergraduate medical curriculum continues to be focused on scientific and technical content. Cited barriers to the integration of humanities include time and space in an already overburdened curriculum, and a lack of consensus on the exact content, pedagogy and instruction. Edmund Pellegrino, physician and scholar of the latter twentieth century, spent much of his professional life promoting the value and importance of the humanities in medical education, seeking the best way to incorporate and teach this content in clinically relevant ways. His efforts included the founding of multiple enterprises starting in the 1960s and 1970s to promote human values in medical education, including the Society for Health and Human Values and its Institute on Human Values in Medicine. Regardless of his efforts and those of many others into the current century, the medical humanities remains a curricular orphan, unable to find a lasting home in medical education and training.

## History of the debate

Debate around the inclusion of humanities in modern American medical education has been ongoing for well over 60 years. While there is much literature over the decades to support the value of humanities content in medical training, such as medical history, bioethics, narrative medicine, medical social sciences, and the arts, its standard inclusion in medical education remains elusive.

Regardless of its purported value to medicine, medical education remains heavily focused on scientific and technical content with little room for the study of disciplines outside of medical science and technology [1–7].^1^ Time and space in an already overburdened curriculum are obvious barriers to inclusion, but questions regarding the type of humanities content, who to teach it, and the most effective methods of teaching remain contentious and unresolved. Edmund Pellegrino, physician and scholar of the latter twentieth century, spent much of his professional life promoting the value and importance of the humanities in medical education, seeking the best way to incorporate and teach humanities content in clinically relevant ways.

Arguments for the inclusion of the humanities in undergraduate medical education center around the contention that medicine and its practice is both a technical, scientific profession, as well as a humanistic, moral one [2, 5].^2^ A profession that must understand not only the scientific basis of disease, and the technology that is available to diagnose and treat disease, but also recognize and appreciate the person in which the disease exists. The argument for incorporating the humanities in medical education is really two fold. First, exposure and investment in humanistic study makes doctors better clinicians. Such study helps doctors understand their patients as whole persons within the context of their lives, and trains them to think critically. Second, the humanities can be a sustaining resource for physicians to maintain their resilience and life balance. Prominent medical educators and scholars, state that humanistic interests such as literature, art and music are potent restorative resources for physicians that can help them to maintain their humanity and perspective through the stresses of medical training and practice [8].

## Value of the humanities to medicine and curricular issues

Training in the humanities provides physicians with the skills to understand, acknowledge and make moral and ethical decisions for their patients as individuals, and to help them understand their patients as members of larger units, such as families and communities [9–11]. Thus, physicians in their training must learn not only the scientific basis of disease but also the personal and human aspects of illness. It is thought that by exposing physicians to training in the humanities they can better learn to ‘see’ their patients and appreciate them as whole persons, to understand their life stories and circumstances, to hone their skills in listening and interpreting their patient’s words, and to read and think more critically [5].^3^ The humanities generally, but more particularly the historical disciplines, are known to provide important tools for critical thinking and inquiry, which are essential to physicians in clinical practice and research [4, 7].^4^ The physician must understand their patients beyond the context of disease and to do so they must have training that goes beyond scientific and technical knowledge and facts.

Melvin Konner in his text, *Becoming a Doctor*, describes his own experiences as an academic anthropologist attending medical school. He reflects on the desires of the American public in relation to their medical treatment and states that most Americans approach medicine as consumers, and as consumers they place a high value on the most up-to-date technologies and scientific knowledge of disease, as do the physicians who serve them. However, Konner states that there is a healing that goes beyond the science of disease and the technology that serves it, and relates to a myriad of nonphysical, existential considerations of the patient that cannot be measured, such as hope, will, courage, heart, mind, and culture. This is the area of medicine that the humanities serve. Physicians need to be trained to appreciate and understand the realm of personhood and culture, as well as the physical, measurable and scientific [12]. To Konner’s point, medical historian John Harley Warner notes that particularly in the latter part of the twentieth century there has been an extraordinary influx of scientific knowledge into the practice of medicine with a popular and professional belief in its efficacy. Yet, this same period does not reflect a consequent improvement in general healthcare [13].

Alvan Feinstein, renowned medical educator, leader and diagnostic innovator of the mid twentieth century warned in 1967 that too great a reliance on scientific knowledge may get in the way of good clinical judgment. An advocate of humanistic training in medical school, Feinstein stated that training in the humanities provided the basic foundation for good patient care. Without this foundation the doctor was no more than, in Feinstein’s words, a “bedside technician” instead of a “scientific healer” [1].^5^ Konner notes that patients believe in science, want science in their care, but science does not completely or even adequately understand or treat their illness [12].^6^ Physician-humanist, Rita Charon, and her colleague Peter Williams state that an education in and appreciation of the humanities provides doctors with the ability “to reach to the heart of human learning about meaning, life and death.” [8].^7^ The humanities provides a foundation for the practice of medicine, the seeing, hearing and healing of the patient.

The second part of the argument for exposing medical students to the humanities is the enriching and restorative qualities that the humanities offer to physicians personally, such as can be gained through the use and exploration of literature and the arts. An introduction to a life of learning, literature, art and music is restorative and can help maintain a life balance, allow resiliency, and sustain the humanity of physicians in the face of dehumanizing pressures and conditions. Humanities exposure and training can be used as a personal resource for physicians to remain balanced, sensitive and understanding of the human condition [5, 8, 10]. Author Robertson Davies in his 1984 lecture at the Johns Hopkins Medical Institute shared his sentiments regarding the importance of humanities in medical education stating that he believed doctors needed to be humanists first and doctors second, as they need to be people with full lives in order to understand the lives others. According to Davies, the humanities, such as art, literature and music, promote a work-life balance and intellectualism that is necessary to survive the daily grind of the physician’s day [14]. Melvin Konner notes that doctors in training learn to become cynical, cold, mechanical, reflexive, and omnipotent over their patients as a way to cope [12].^8^ It is believed that exposure to the humanities can be used to help counter these negative emotions and attitudes. Edmund Pellegrino wrote that engagement in the humanities offers three important benefits to physicians that are essential to their competence: methods of inquiry or thought, content of knowledge, and also the power to feed and invigorate the spirit [5].

Pellegrino emphasized that physicians must not only be knowledgeable and capable of using current scientific technologies, but also humane clinical caregivers even to the point of protecting their patients against the dehumanization of technology and modern medicine. He believed that integrating humanities within professional medical education, as opposed to relying on material taught in a baccalaureate liberal arts degree, is the best way to stimulate the minds of new physicians and humanize their medical practice [5].^9^

Science and technology teach physicians how to treat disease, not how to help patients live with chronic, debilitating illness, or how to die. Doctors must treat the spiritual, emotional and physical aspects of their patients, and exposure and experience with the humanities help doctors respond to the human needs of medicine [10, 15, 16]. Physician author Abraham Nussbaum in his recent book entitled *The Finest Traditions of My Calling: One Physician’s Search for the Renewal of Medicine*, Nussbaum provides reflections of his own experience as a medical student and practicing physician. He describes a “confusion in medicine”. He states “the units shine, the surgical instruments are precise, the medical techniques are novel, and yet the physicans are discouraged.” [17].^10^ Physicians have lost their sense of fulfillment from their profession and life as a healer. Nussbaum calls for a renewal of medicine in the twenty-first century with a greater integration and presence of humanism in the profession and its training [17].

While teaching humanities content is being promoted as a solution to the reductionism of modern medicine, the use of humanities in medical education has a long history in American medical education. Abraham Flexner’s 1910 assessment of medical schools in the US and Canada dramatically reformed medical education into an academic, laboratory science based education [18]. Flexner assumed that the humanities would be taught as way of promoting an educated class of professionals [19]. Other early to mid-twentieth century leaders in academic medicine, such as Sir William Osler, Henry Sigerist, and Owsei Temkin saw value in and used humanities knowledge and methods to bring greater context and meaning to their clinical practices and research [20, 21]. Osler frequently used biography as a method of teaching professionalism, medical ethics and values to the young physicians that he taught [22]. Henry Sigerist, Osler’s professional successor at Johns Hopkins, valued medical history as a critical medical field of knowledge and study, and used it in medical education as a way of displaying the errors of past thought and lessons to be learned through historical insight [13, 21]. Temkin, Sigerist’s student, used history in medical education as a tool of professional development and grounding for students [6, 23].

Through the twentieth century, the burgeoning amount of science and technology available to medicine has pushed humanities out of the curriculum. In the wake of 1960s social activism, civil unrest and the exposure of medical research atrocities, a renewed cry and realization of the need for humanistic training in medicine became apparent. The Society for Health and Human Values initiated in 1969 under the leadership and guidance of Edmund Pellegrino became one of the driving forces for reintegrating humanistic content back into medical education. Created and lead by humanist physicians and humanities scholars, the Society promoted humanities training and scholarship in medical education and practice.^11^ The Society’s Institute on Human Values in Medicine funded by the National Endowment for the Humanities created multiple pathways to understand how the humanities could be taught in medical schools and to provide the curricular resources to medical schools to start medical humanities courses and programs of study. The Institute held workshops, conferences and focus groups directed at examining, providing and promoting the integration of humanities disciplinary content into the standard medical curriculum [8, 24].

As a result of such efforts the use of humanities in the limited and isolated form of medical ethics became and is currently a required part of the standard medical school, owing in large part to the value physician-educators have given to it [21]. The Association of American Medical Colleges (AAMC), the professional association of the medical education community, has published standards that require all medical schools to incorporate training that assures students and graduates will have a working knowledge of medical ethics and ethical decision-making in caring for patients and families [25]. However, academic physicians and non-physicians are critical of this content. A survey issued across US and Canadian medical schools in 2000 found the ethics training incorporated into curricula to be highly variable, providing inconsistent value to training [26]. Cooter states that current instruction generally lacks historical, social and cultural input, making it void of context, and thus serves only to validate itself and current systems, adding nothing to critical thinking and analysis [27, 28]. Evidence of the success of current ethics training in medical schools remains suspect and questionable. The models and modes of ethics training continue to be discussed and evaluated.^12^

Humanist physicians and medical educators in the twenty-first century continue to see a need for rebalancing the medical curriculum between science and humanities material. Current day efforts are reminiscent of Pellegrino’s Institute on Human Values in Medicine. Some of these efforts, such as PRIME – *Project to Rebalance and Integrate Medical Education* – see the humanities as a necessary element of teaching professionalism in medical education and residency training. The organizers of PRIME, some of which were mentees of Edmund Pellegrino and trainees of the Institute on Human Values in Medicine, work toward the same goal as Pellegrino and the Institute – examination of the best methods to integrate humanities into medical training and education. They call for a return to Flexner’s ideal of a well-educated humanist profession [29, 30]. Others, in the wake of the centennial of Flexner’s 1910 report, also call for major revisions of medical curricula in areas outside of the biosciences that promote greater competency in critical analysis, habits of inquiry, ethical conduct, communications and cultural competency [31, 32]. The ‘medical humanities’ and disciplines such as the behavioral sciences are being looked upon to provide such competency [8, 21].

## In sum …

The value of humanities and humanistic inquiry is not publically challenged, yet how to fit it into an overloaded curriculum remains a conundrum.^13^ Medical educators interested and dedicated to incorporating humanities into their curricula struggle with questions of content, methods, type of integration and teaching structures. Physician scholars and educators such as Edmund Pellegrino and Rita Charon suggest that the humanities be team taught by faculty experts in the various fields, such as philosophy, history, literature, theology and ethics, visual arts and jurisprudence so as not to lose the intellectual essence, culture and rigor of each [2, 8]. However, forums of instruction that combine so many areas of study face the danger of diluting the relevance of each subject, losing their pedagogical depth and impact [21]. Physician-scholars have reported discontinuing humanities courses in medical school because the content was too limited, and the student body was not adequately prepared for critical analysis [33]. A survey of medical school faculty in the United States that teach medical humanities in their schools advocate for the breakdown of disciplinary boundaries and support methods that integrate humanities disciplines in ways that follow their own theories and norms yet juxtapose them against each so as to create a dialogue, a so called “informed disciplinarity”, “synthetic interdisciplinarity” or “transdisciplinarity” [34].^14^

Physician historians and academic historians, such as Howard Kushner, continue to embrace the importance of teaching medical history as a tool [4, 6]. One such physician historian and medical educator, Jacalyn Duffin, is a strong advocate of such coursework and content. She has developed her own curriculum that integrates history of medicine into each basic science and clinical competency area across all four years of the curriculum in her Canadian medical school. Her goals in teaching this curriculum are to foster skepticism, create humility, and embed a habit and interest in lifelong learning in each student. The success of this curriculum has gained her further access to both increased curricular time and faculty involvement [3]. Case based methods have also been advocated for teaching medical students who are otherwise inundated with didactic facts. Methods based on concrete examples in clinical practice communicate information more effectively in an active rather than passive experience [33]. The Department of Medical Humanities at the Penn State College of Medicine, a department founded by a contemporary of Edmund Pellegrino and a leader within his Institute on Human Values in Medicine, continues to offer a rich and multi-disciplinary humanities curriculum within their medical school. In addition, this school spearheads a curriculum on Health Systems Science, which incorporates population health, policy, medical systems, leadership and assessment of quality care, areas which may relate to social and human aspects of care. Such novel curricula suffer the same challenges previously outlined for the humanities: challenges of space in the curriculum, value and who should teach it [35].^15^

It is important to note that teaching the humanities is not seen as an assurance of creating humanitarianism in physicians, like a magic bullet. It is seen, instead, as a way of developing, encouraging, stimulating and motivating such emotions and practices within medical practitioners [36]. Even the most passionate proponents of humanities training in medical education realize that the drive for service and humanism in medicine is distinct from anything that can be taught. However, the demands of medical education and clinical training have been seen to drive out these beliefs, leaving trainees cynical, mechanical, entitled and unable or unwilling to connect with their patients. It is hoped that the humanities can move students to see beyond the grind of long hours, overwhelming clinical services, patient complexities and corporatized medicine to appreciate their patients in their lives and continuously restore their own personal sense of purpose and self [5, 36, 37].

Edmund Pellegrino refers to medicine as “the most humane of sciences, the most empiric of arts, and the most scientific of humanities” [5].^16^ He asks, “Can the doctor simultaneously attend Man the molecular aggregate and Man the person; Man the unit of a complex society and Man the ineffable?” [2].^17^ The methods to train such a physician remain in question and in development with as much question today as when Pellegrino and the Society for Health and Human Values began their efforts almost fifty years ago. The medical humanities remains the curricular orphan. Can it find a home in medical education? Can it answer medicine’s cry for health and renewal?

## Footnotes

1 Discussion of the value of relevant humanities disciplines in medical education can be found through the decades. Alvan R. Feinstein, *Clinical Judgement* (Baltimore: The Williams & Wilkins Company, 1967). Edmund D. Pellegrino, *Humanism and the Physician* (Knoxville: University of Tennessee Press, 1979), Philosophy of medicine. Jacalyn Duffin, “Infiltrating the Curriculum: An Integrative Approach to History for Medical Students,” *Journal of Medical Humanities* 16 (1995). Howard I. Kushner, “History as a Medical Tool,” *Lancet* 371, no. February 16 (2008). Howard I. Kushner, Leslie S. Leighton, “The Histories of Medicine: Toward an Applied History of Medicine,” in *Humanities in the Twenty-First Century: Beyond Utility and Markets*, ed. Anna Upchurch Eleonora Belfiore (London: Palgrave, MacMillan, 2013); Edmund D. Pellegrino, *The Philosophy of Medicine Reborn: A Pellegrino Reader*, ed. Jr. and Fabrice Jotterand H. Tristram Englehardt, Notre Dame Studies in Medical Ethics (Notre Dame, Indiana: University of Notre Dame Press, 2008); David S. Jones, Jeremy A. Greene, Jacalyn Duffin, and John Harley Warner, “Making the Case for History in Medical Education,” *Journal of the History of Medicine and Allied Sciences* (2015).

2 Pellegrino wrote extensively on this topic. A synopsis of these views can be found in his 1979 text, *Humanism and the Physician,* as well as his 2008 text, *The Philosophy of Medicine Reborn: A Pellegrino Reader*.

3 Pellegrino, *The Philosophy of Medicine Reborn: A Pellegrino Reader*. See page 338.

4 The value of historical training to physicians is documented and summarized in the work of Howard Kushner, “History as a Medical Tool,” *Lancet* 371, no. February 16 (2008). Also see the Jones and colleagues 2015 paper “Making the Case for History in Medical Education”.

5 Feinstein. Page 363.

6 See Konner’s final chapter.

7 Rita Charon, Peter Williams, “Introduction: The Humanities and Medical Education,” *Academic Medicine* 70, no. 9 (1995). Page 758.

8 See Konner, pages 361–377.

9 Pellegrino, *The Philosophy of Medicine Reborn: A Pellegrino Reader*. See page 338

10 Abraham M. Nussbaum, *The Finest Traditions of My Calling: One Physician’s Search for the Renewal of Medicine* (New Haven: Yale University Press, 2016). Page 7.

11 The American Society for Bioethics and Humanities (ASBH) was founded in January 1998 through the consolidation of three former associations in the field: the Society for Health and Human Values (SHHV), the Society for Bioethics Consultation (SBC), and the American Association of Bioethics (AAB). - See more at: http://www.asbh.org/about/history/index.html#sthash.GXbSGAnK.dpuf

12 Data indicating the failure to increase the ethical behavior of medical students with a new cultural competency based ethics training program was presented in a panel forum during the 2010 Association of American Medical Colleges (AAMC) *Group on Graduate Research, Education and Training (GREAT) Annual Meeting*, October 21–23, 2010. Michael D. Mumford, then George Lynn Cross Distinguished Research Professor of Psychology, University of Oklahoma presented this data as part of a panel presentation entitled “Models of RCRS (Responsible Conduct of Research Scholarship) Training” at the Astor Crowne Plaza Hotel, New Orleans, Louisiana on October 23, 2010.

13 This question remains across decades from Pellegrino’s work in the 1970s to Jones and colleagues in 2015.

14 Wear, Pages 216–17.

15 Personal communications and interviews with current and past department faculty of the Penn State College of Medicine and leadership staff of the Milton S. Hershey Medical Center.

16 Pellegrino, *The Philosophy of Medicine Reborn: A Pellegrino Reader*. Page 309.

17 Pellegrino, *Humanism and the Physician*. Page 16.
